# Protein *O*-GlcNAcylation as a nutrient sensor signaling placental dysfunction in hypertensive pregnancy

**DOI:** 10.3389/fendo.2022.1032499

**Published:** 2022-12-01

**Authors:** Rinaldo Rodrigues dos Passos Junior, Raiany Alves de Freitas, Vanessa Dela Justina, Sebastián San Martín, Victor Vitorino Lima, Fernanda Regina Giachini

**Affiliations:** ^1^ Graduate Program in Biological Sciences, Federal University of Goiás, Goiânia, Brazil; ^2^ Institute of Biological and Health Sciences, Federal University of Mato Grosso, Barra do Garças, Brazil; ^3^ Biomedical Research Center, School of Medicine, Universidad de Valparaíso, Valparaíso, Chile

**Keywords:** hypertension, placenta, *O*-GlcNac, glycosylation, glucose uptake, fetal growth restriction

## Abstract

**Introduction:**

During pregnancy, arterial hypertension may impair placental function, which is critical for a healthy baby's growth. Important proteins during placentation are known to be targets for O-linked β-N-acetylglucosamine modification (O-GlcNAcylation), and abnormal protein O-GlcNAcylation has been linked to pathological conditions such as hypertension. However, it is unclear how protein O-GlcNAcylation affects placental function and fetal growth throughout pregnancy during hypertension.

**Methods:**

To investigate this question, female Wistar and spontaneously hypertensive rats (SHR) were mated with male Wistar rats, and after pregnancy confirmation by vaginal smear, rats were divided into groups of 14, 17, and 20 days of pregnancy (DOPs). On the 14th, 17th, and 20th DOP, rats were euthanized, fetal parameters were measured, and placentas were collected for western blot, immunohistochemical, and morphological analyses.

**Results:**

SHR presented a higher blood pressure than the Wistar rats (p=0.001). Across all DOPs, SHR showed reduced fetal weight and an increase in small-for-gestational-age fetuses. While near-term placentas were heavier in SHR (p=0.006), placental efficiency decreased at 17 (p=0.01) and 20 DOPs (p<0.0001) in this group. Morphological analysis revealed reduced junctional zone area and labyrinth vasculature changes on SHR placentas in all DOPs. O-GlcNAc protein expression was lower in placentas from SHR compared with Wistar at 14, 17, and 20 DOPs. Decreased expression of O-GlcNAc transferase (p=0.01) and O-GlcNAcase (p=0.002) enzymes was found at 14 DOPs in SHR. Immunohistochemistry showed reduced placental O-GlcNAc content in both the junctional zone and labyrinth of the placentas from SHR. Periodic acid-Schiff analysis showed decreased glycogen cell content in the placentas from SHR at 14, 17, and 20 DOPs. Moreover, glucose transporter 1 expression was decreased in placentas from SHR in all DOPs.

**Conclusions:**

These findings suggest that decreased protein O-GlcNAcylation caused by insufficient placental nutritional apport contributes to placental dysfunction during hypertensive pregnancy, impairing fetal growth.

## Introduction

Hypertensive disorders of pregnancy (HDPs) are a worldwide health problem that complicate up to 10% of all pregnancies and are among the leading causes of pregnancy-related mortality and morbidity, with an estimated 14% of global pregnancy deaths (1, 2). Therefore, the term HDP is commonly used to describe a wide spectrum of patients, including those with mildly elevated blood pressure, as well as those with severe hypertension, with or without organ dysfunction (3). Although many pregnancies affected by hypertension usually progress well and have normal outcomes, there is an increased risk of complications such as preeclampsia, fetal growth restriction, and perinatal death (4). Despite various etiopathologies, HDPs are characterized by structural and functional alterations of the placenta (5, 6).

The placenta is undoubtedly known to be vital during pregnancy because it helps to establish the pregnant state, protect the embryo, and promote the exchange of nutrients, gasses, and waste products so that the embryo can survive and develop in the intrauterine environment (7). During the multiple events of placental development, several signaling pathways are triggered to coordinate these processes, and specific proteins that regulate placental function are known to be targets for post-translational modification involving glycans (8).

*O*-linked β-N-acetylglucosamine modification (*O*-GlcNAcylation) is a dynamic and reversible process that regulates protein stability, activity, and localization, and therefore cellular response, by the addition of a single saccharide to the serine, threonine, and tyrosine sites of nuclear, cytosolic, and mitochondrial proteins (9). The cycling of *O*-GlcNAcylation is tightly controlled by two unique and essential enzymes: *O*-GlcNAc transferase (OGT) and *O*-GlcNAcase (OGA), which catalyze the addition and removal of *O*-GlcNAc, respectively (10). The substrate for *O*-GlcNAcylation requires glucose conjugation with amino acids, lipids, and nucleic acids to produce uridine diphosphate-N-acetylglucosamine (UDP-GlcNAc) through the hexosamine biosynthetic pathway (HBP) (11). Therefore, HBP and *O*-GlcNAcylation are considered to be major nutrient-sensitive pathways (12, 13) and aberrant *O*-GlcNAcylation has been associated with metabolic disruption and pathological conditions, such as diabetes and arterial hypertension (14, 15).

The growth and development of a healthy baby require the transport of essential nutrients through the placenta, such as glucose and amino acids. Glucose reaches the growing fetus *via* numerous glucose transporters (GLUT) present in the placenta, where GLUT1 and GLUT3 isoforms are known to be major contributors to placental glucose transport (16, 17). Moreover, nutrient uptake in response to fetal demand is tightly coordinated by an array of signaling pathways, and *O*-GlcNacylation is known to be a nutrient-sensing pathway involved in glucose utilization (12, 13). Interestingly, arterial hypertension during pregnancy has been associated with impaired fetal growth and small-term babies. However, the involvement of the *O*-GlcNacylation pathway on placental nutritional apport and fetal development in this condition remains unknown.

Therefore, in the present study, we investigated how hypertension affects *O*-GlcNacylation of proteins, availability of glucose in the placenta, and fetal growth during pregnancy in hypertensive rats.

## Materials and methods

### Ethics statement

All the procedures and animal handling and maintenance were carried out according to the guidelines provided by the Brazilian College of Animal Experimentation upon approval by the Ethics Committee on the Use of Animals of the Federal University of Mato Grosso (CEUA-Araguaia; #23108.038471/2019-14).

### Animals

Female Wistar and Spontaneously Hypertensive Rats (SHR) (12-14 weeks old, 180-200 g) obtained from the Laboratory of Vascular Biology and Histopathology of the Institute of Biological and Health Sciences at the Federal University of Mato Grosso were used in this study. The rats were maintained in the animal facility room, at 23 ± 2°C, with 12-hour light/dark cycles, fed a standard commercial diet, and received free water intake. The blood pressure was measured by tail-cuff plethysmography after three days of adaptation to the device, before mating.

### Mating and pregnancy determination

For mating, females were housed with males of the same species during the night (of ±4 females for each male). By the morning of the following day, the rats were separated, and vaginal smears were taken to observe the presence of sperm and keratinized cells from the estrous cycle. If positive for the presence of spermatozoa, this was designated gestational day 0.

### Experimental design

Pregnant SHR and Wistar rats were separated into hypertensive and normotensive groups, respectively, and divided into 14, 17, and 20 days of pregnancy (DOP) subgroups (n = 6, for each group). On the 14^th^, 17^th^, and 20^th^ DOP, rats were anesthetized with 3% sodium pentobarbital (50 mg/kg body weight, i.p.) and submitted to laparotomies for removal of the placentas and fetuses. The placentas were cleaned of connective tissue, cut in half, and stored at -80°C or immersed in a fixative solution for the histological experiments. The living fetuses were individually weighted and classified according to the mean values of fetal weights in the normotensive group as small for gestational age [(SGA) fetal weight < Wistar mean - SD x 1.7]; appropriate for gestational age [(AGA) fetal weight within Wistar mean ± SD x 1.7]; and large for gestational age [(LGA) fetal weight > Wistar mean +SD x 1.7] (18), demised fetuses were not included. Posteriorly, rats were killed by pneumothorax, and fetuses were killed by placement in a CO_2_ chamber.

### Western blotting

Placentas were immersed in liquid nitrogen and mechanically macerated to obtain total cell lysate by incubating samples with lysis buffer containing protease inhibitors. Protein concentration was determined using the Bradford Assay Kit (Sigma-Aldrich). Extracted proteins (60 μg per lane) were loaded and separated on a polyacrylamide gel (8-10%) by electrophoresis and transferred to a nitrocellulose membrane (Sigma-Aldrich). The success of protein transfer was further detected by Ponceau S staining. Non-specific binding sites were blocked with 5% skimmed dry milk in Tris-buffered saline solution with Tween-20 (TBS-T, pH 7.6) for 1 hour, at room temperature. Membranes were rinsed and incubated with primary antibodies overnight at 4°C under constant agitation. The following antibodies were used: anti-*O*-GlcNAc (Sigma-Aldrich Cat# O7764, RRID : AB_1079524, 1:500), anti-OGT (Abcam Cat# ab50273, RRID : AB_881784, 1:1000), anti-OGA (Sigma-Aldrich Cat# SAB4200311, RRID : AB_10898726, 1:500), anti-GLUT1 (Abcam Cat# ab115730, RRID : AB_10903230, 1:8000), anti-β-actin (Abcam Cat# ab8227, RRID : AB_2305186, 1:3000). Thereafter, membranes were removed from primary antibodies and washed with TBS-T. Membranes were treated with the respective secondary antibodies for 1 hour at room temperature. Protein bands were detected using the ECL Plus Western Blotting Detection System (GE Healthcare) and then quantified using an image-analysis software program. The protein expressions were normalized to the intensity of β-actin protein and were further expressed as arbitrary units.

### Histological procedures

Placentas were fixed in methacarn solution (60% methanol, 30% chloroform, and 10% acetic acid), for 3 hours, at 4 °C, under constant agitation. Then, the placentas were dehydrated in successive alcohol dilutions, clarified in xylene, and subsequently infiltrated with paraffin. Sections of 4 µm thickness were made using a microtome, stretched in a floating bath at 50°C, and adhered to glass slides previously treated with poly-*L*-lysine 0.1% (Sigma) for better adhesion of the sections.

### Hematoxylin and eosin staining

For morphological analysis, hematoxylin-eosin staining was performed. Sections were deparaffinized and rehydrated, and the slides were immersed in hematoxylin for 1 minute, rinsed in running water and in distilled water, and subsequently counterstained with aqueous eosin for 30 seconds.

### Periodic Acid-Schiff (PAS) staining

Sections were deparaffinized and rehydrated. Slides were dipped in a periodic acid solution and rinsed in distilled water. Subsequently, slides were placed in Schiff’s solution (basic fuchsin, sodium bisulfite, hydrochloric acid, and distilled water) at room temperature and rinsed in sulfuric water. Finally, the histological sections were counterstained with Harris hematoxylin to fully recognize PAS-positive staining and rinsed well in distilled water.

### Immunohistochemistry

Immunohistochemistry was performed according to a previously established protocol (19). Sections were deparaffinized and rehydrated. Each of the following steps was followed by rinses in 0.1M phosphate buffer solution (PBS) – pH 7.2-7.4. Antigenic epitope retrieval was performed by immersing slides in citrate buffer (pH 6.0) at 95°C for 25 minutes. Then, sections were incubated in a hydrogen peroxide solution (3% H_2_O_2_ (v/v) in PBS) to block endogenous peroxidase activity. To reduce nonspecific antigenic sites (background), the slides were incubated with Cas-Block solution (ThermoFisher Scientific), for 30 minutes. The incubation of the primary antibody was preceded by a series of standardizations, and the antibody dilutions were previously determined. The slides were subsequently incubated with the anti-*O*-GlcNAc primary antibody (Cell Signaling Technology Cat# 9875, RRID : AB_10950973), diluted 1:50 in PBS containing 0.3% (v/v) Tween 20, overnight at 4°C in a humid chamber. After extensive rinsing in PBS, all sections were incubated with biotin-conjugated goat anti-mouse IgG (Sigma-Aldrich Cat# A9044, RRID : AB_258431) diluted 1:250 in PBS for 1 h, at room temperature. The peroxidase reaction was visualized using the NovaRED^®^ kit (Vector). A slight counter-stain was performed with Harris hematoxylin to provide a contrast to the chromogen. The secondary antibody specificity was tested by omitting the primary antibody. The specificity of the primary antibody was tested in experiments with positive control tissues, already described in the literature (19).

### Histochemical, morphological, and morphometric analysis

Sections were examined in a Nikon Eclipse microscope, and the images were captured using a digital camera (Opton) and TCapture software. For morphometric analyses, the areas of the junctional zone and the labyrinth were measured in (mm^2^) using the Image-Pro-Plus software (Media Cybernetics, Silver Spring, MD, USA). PAS stain-positive cells were counted for each captured field and normalized by the area (mm^2^) of the junctional zone. They were expressed as the number of cells per mm^2^ of the junctional zone. Protein expression of immunohistochemistry staining was determined by semi-quantitative analysis using ImageJ Fiji (WS Rasband, National Institute of Health, Bethesda, MD) and was expressed as the percentage of staining intensity normalized by the nuclei number, as described previously (20).

### Statistical analysis

Data were presented as mean ± standard error of the mean (SEM), and “n” represents the number of animals used in the experiment. Statistical analysis was performed using the Prisma program (GraphPad Prism 5.0, GraphPad Software Incorporated, CA) (GraphPad Software Inc.), with Student t test, compared to the respective normotensive group. For analysis between three or more groups, a one-way analysis of variance (One-Way ANOVA) followed by the Tukey post-test was used. For percentage analysis, Fisher’s exact test was used in the SPSS program (IBM SPSS Statistics 20). P values <0.05 were considered statistically significant.

## Results

### Reduced placental *O*-GlcNac, OGT, and OGA expression in SHR

**Figure 1:**
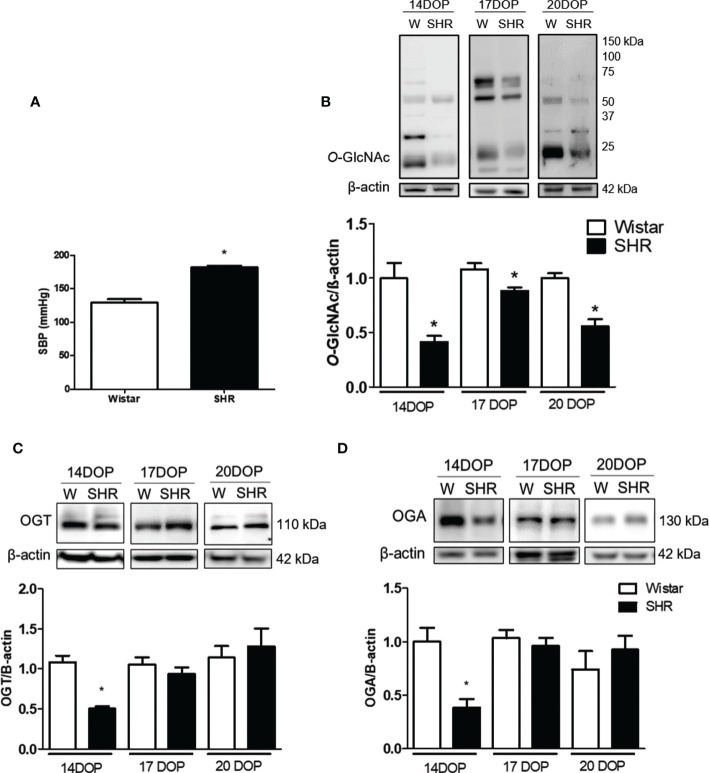
Reduced placental *O*-GlcNAc, OGT and OGA expression in SHR. **(A)** Bar graph showing SBP (mmHg) in Wistar and SHR; n = 6 each group. **(B)** Upper representative picture of western blot membrane of placental *O*-Glcnac expression in Wistar and SHR at 14, 17 and 20 DOP. Bar graph showing the *O*-Glcnac expression in Wistar and SHR at 14, 17 and 20 DOP; n = 6 each group. **(C)** Upper representative picture of western blot membrane of placental OGT expression in Wistar and SHR at 14, 17 and 20 DOP. Bar graph showing the OGT expression in Wistar and SHR at 14, 17 and 20 DOP, n = 6 each group. **(D)** Upper representative picture of western blot membrane of placental OGA expression in Wistar and SHR at 14, 17 and 20 DOP. Bar graph showing the OGA expression in Wistar and SHR at 14, 17 and 20 DOP, n = 6 each group. Values are presented as means ± SEM, and data were analyzed by one‐way ANOVA, followed by Tukey post-test. *p < 0.05 vs Wistar at respective DOP. Protein expression was individually determined and corrected by β−actin expression.

Systolic blood pressure (SBP) was higher in SHR when compared to Wistar rats [(mmHg) 181 ± 3 vs. 128 ± 5 Wistar; p < 0.001] (**Figure 1A**). We accessed the *O*-GlcNac protein expression on placentas from SHR and Wistar rats at 14, 17, and 20 DOP. Placentas from SHR presented reduced *O*-GlcNac protein expression at 14, 17, and 20 DOP when compared to those from Wistar rats (**Figure 1B**). Moreover, reduced expression of OGT (0.5 ± 0.01 vs. 0.9 ± 0.1 Wistar; p = 0.01) and OGA (0.4 ± 0.01 vs. 1 ± 0.1 Wistar; p = 0.002) enzymes was found on 14 DOP in SHR (**Figures 1C, D**), but not in 17 or 20 DOP.

### Reduced fetal weight and increased percentage of small for gestational age (SGA) fetuses in SHR

Fetal parameters from pregnant SHR and Wistar rats at 14, 17, and 20 DOP are shown in **Table 1**. SHR presented increased pre-implantation losses. Reduced fetal weight with an increased number of small for gestational age (SGA) fetuses was found on SHR at 14, 17, and 20 DOP when compared to Wistar. The percentage of SGA fetuses increased throughout pregnancy in SHR, and near term, 100% of all the fetuses were found to be SGA in this group.

**Table 1:** Fetal parameters from Wistar and SHR at 14, 17 and 20 DOP.

	14 DOP		17 DOP		20 DOP	
	Wistar	SHR	Wistar	SHR	Wistar	SHR
**Pre-implantation loss**	1.2 ± 0.4	4 ± 0.7*	1 ± 0.6	4 ± 0.8*	0.5 ± 0.2	4 ± 1.7*
**Fetal weight (g)**	0.15 ± 0.004	0.13 ± 0.007*	0.9 ± 0.03	0.7 ± 0.03*	4.9 ± 0.1	3.4 ± 0.04*
**SGA (%)**	3.40	27.60*	1.60	43.50*	1.50	100.0*
**AGA (%)**	96.60	72.40*	91.80	56.50*	97.0	0.0*
**LGA (%)**	0.0	0.0	6.60	0.0	1.5	0.0

SGA, small for gestational age; AGA, appropriate for gestational age; LGA, large for gestational age. *p<0.05 vs Wistar. Student unpaired t-test and Fisher exact test (%).

### Placental structural and functional alterations in SHR

Once the fetal weight was decreased and the fetuses were smaller in SHR, we investigated the placenta (**Table 2**). Placental weight was found to increase throughout pregnancy, and near-term placentas from SHR were found to be significantly heavier when compared to those from Wistar rats [(g) 0.6 ± 0.02 vs. 0.4 ± 0.02 Wistar; p = 0.006]. On the other hand, placental efficiency, indicated by the fetal/placental weight ratio, decreased in SHR at 17 (1.7 ± 0.1 vs. 2.3 ± 0.1 Wistar; p=0.01) and 20 DOP (6 ± 0.2 vs. 10 ± 0.2 Wistar; p<0.0001).

**Table 2:** Placental parameters from Wistar and SHR, at 14, 17 and 20 DOP.

	14 DOP		17 DOP		20 DOP	
	Wistar	SHR	Wistar	SHR	Wistar	SHR
**Placental weight (g)**	0.16 ± 0.01	0.17 ± 0.01	0.37 ± 0.03	0.42 ± 0.02	0.45 ± 0.007	0.55 ± 0.02*
**fetal/placental** **weight ratio**	0.98 ± 0.09	0.78 ± 0.06	2.33 ± 0.1	1.78 ± 0.1*	10.43 ± 0.2	6.1 ± 0.2*

*p<0.05 vs Wistar. Student unpaired t-test.

**Figure 2:**
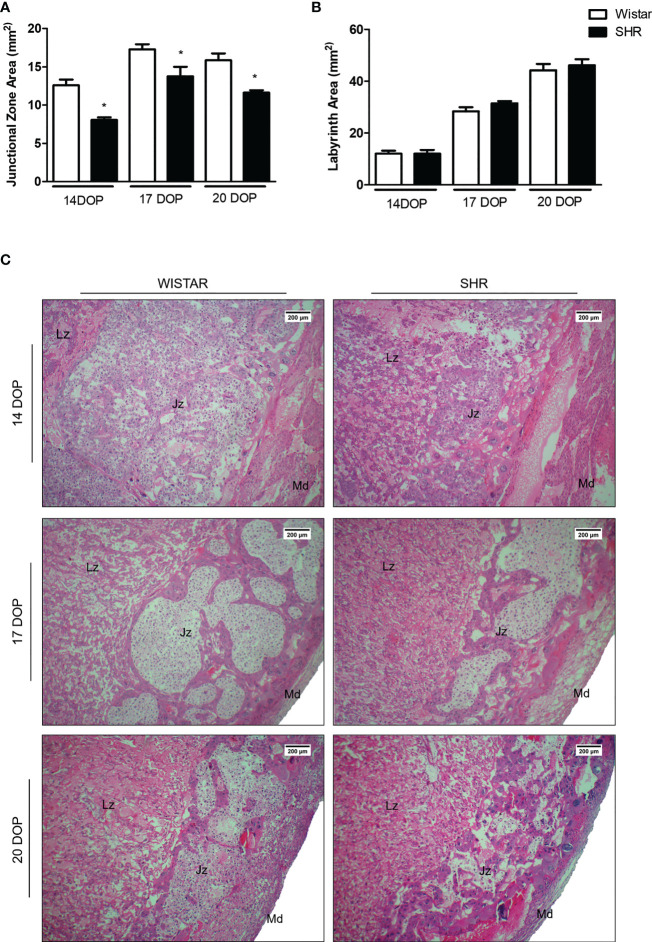
Morphometric and morphological alterations in placentas from SHR. **(A)** Bar graph showing the Jz area (mm^2^) of placentas from Wistar and SHR at 14, 17 and 20 DOP; n = 5-6 each group. **(B)** Bar graph showing the Lz (mm^2^) area of placentas from Wistar and SHR at 14, 17 and 20 DOP; n = 5-6 each group. **(C)** Hematoxylin & eosin-stained placentas (40X) from SHR and Wistar at 14, 17 and 20 DOP; Overall view of the labyrinth (Lz) region, junctional zone (Jz) and maternal decidua (Md). Values are presented as means ± SEM, and data were analyzed by Student unpaired t-test. *p < 0.05 vs Wistar at respective DOP.

Morphometric analysis showed that the junctional zone (Jz) increases throughout pregnancy, reaching a higher peak area on day 17 of pregnancy in both Wistar and SHR. However, placentas from SHR presented decreased Jz area on all DOP analyzed, compared to the respective Wistar group (**Figures 2A–C**). Although no alterations were found in the labyrinth zone (Lz) between groups (**Figure 2B**), the placental Lz from SHR presented a compacted area with thicker wall vessels and vascular congestion (**Figure 2C**).

### Reduced protein *O*-GlcNAcylation on both Jz and Lz of the placentas from SHR

**Figure 3:**
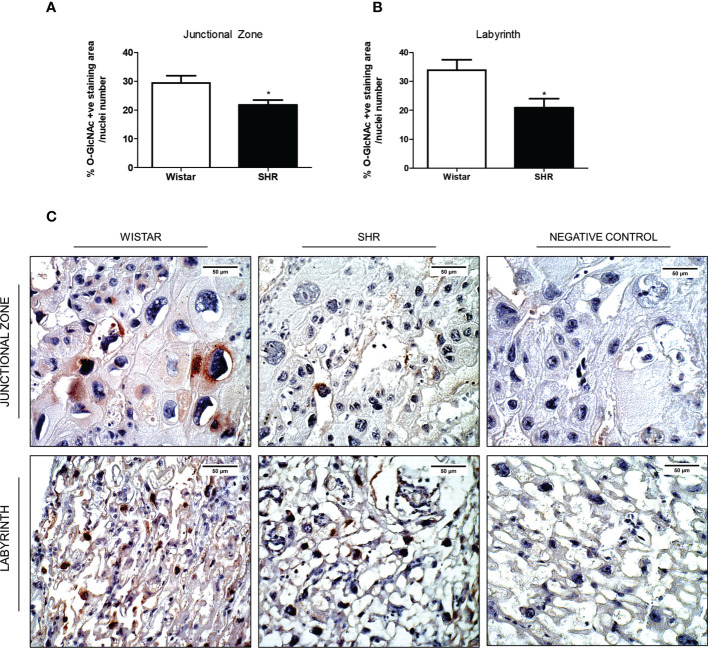
Reduced *O*-GlcNAc protein expression on both Jz and Lz of the placentas from SHR. **(A)** Bar graph showing the % of *O*-GlcNAc protein expression on the Jz of the placentas from Wistar and SHR; n = 4 each group. **(B)** Bar graph showing the % of *O*-GlcNAc protein expression on the Lz of the placentas from Wistar and SHR; n = 4 each group. **(C)** Immunoreaction on the Jz and Lz of the placentas from Wistar and SHR. After antigen retrieval, sections were treated with anti-*O*-GlcNAc (1:50) and biotin-conjugated goat anti-mouse IgG (1:250). Negative control sections were incubated with PBS or with the secondary antibody (omitting the primary antibody). Values are presented as means ± SEM, and data were analyzed by Student unpaired t-test. *p < 0.05 vs Wistar.

We performed immunohistochemistry to characterize the localization of the *O*-GlcNAc protein profile in placental tissue. Immunohistochemistry analysis showed reduced *O*-GlcNAc deposition on both Jz and Lz of the placentas from SHR when compared to Wistar rats (**Figures 3A, B**). In these zones, proteins from both endothelial and trophoblast cells were the most frequent targets for *O*-GlcNAcylation (**Figure 3C**).

### Reduced glucose storage and GLUT1 expression on placentas from SHR

**Figure 4:**
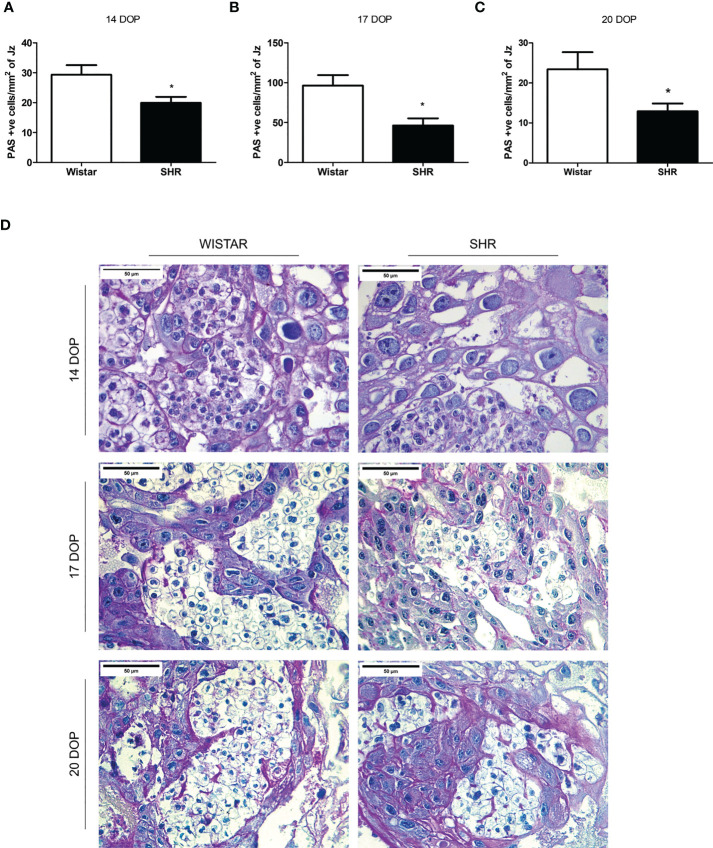
Reduced glycogen cells content in placentas from SHR at 14, 17 and 20 DOP. **(A-C)** Bar graph showing the placental amount of glycogen cells per mm2 of Jz in Wistar and SHR at 14, 17 and 20 DOP, respectively. **(D)** Representative placental tissue sections showing PAS positive stained glycogen cells in Wistar and SHR at 14, 17 and 20 DOP. Values are presented as means ± SEM, and data were analyzed by Student unpaired t-test. *p < 0.05 vs Wistar at respective DOP.

Thus, we decided to investigate placental glucose uptake and storage. PAS analysis evidenced the presence of islets of glycogen cells in the Jz of placentas from both groups, with a greater amount of glycogen cells at 17 DOP when compared to the other gestational time points. We observed a decreased glycogen cell content in placental tissue from SHR at 14, 17, and 20 DOP when compared to Wistar (**Figures 4A–D**).

**Figure 5:**
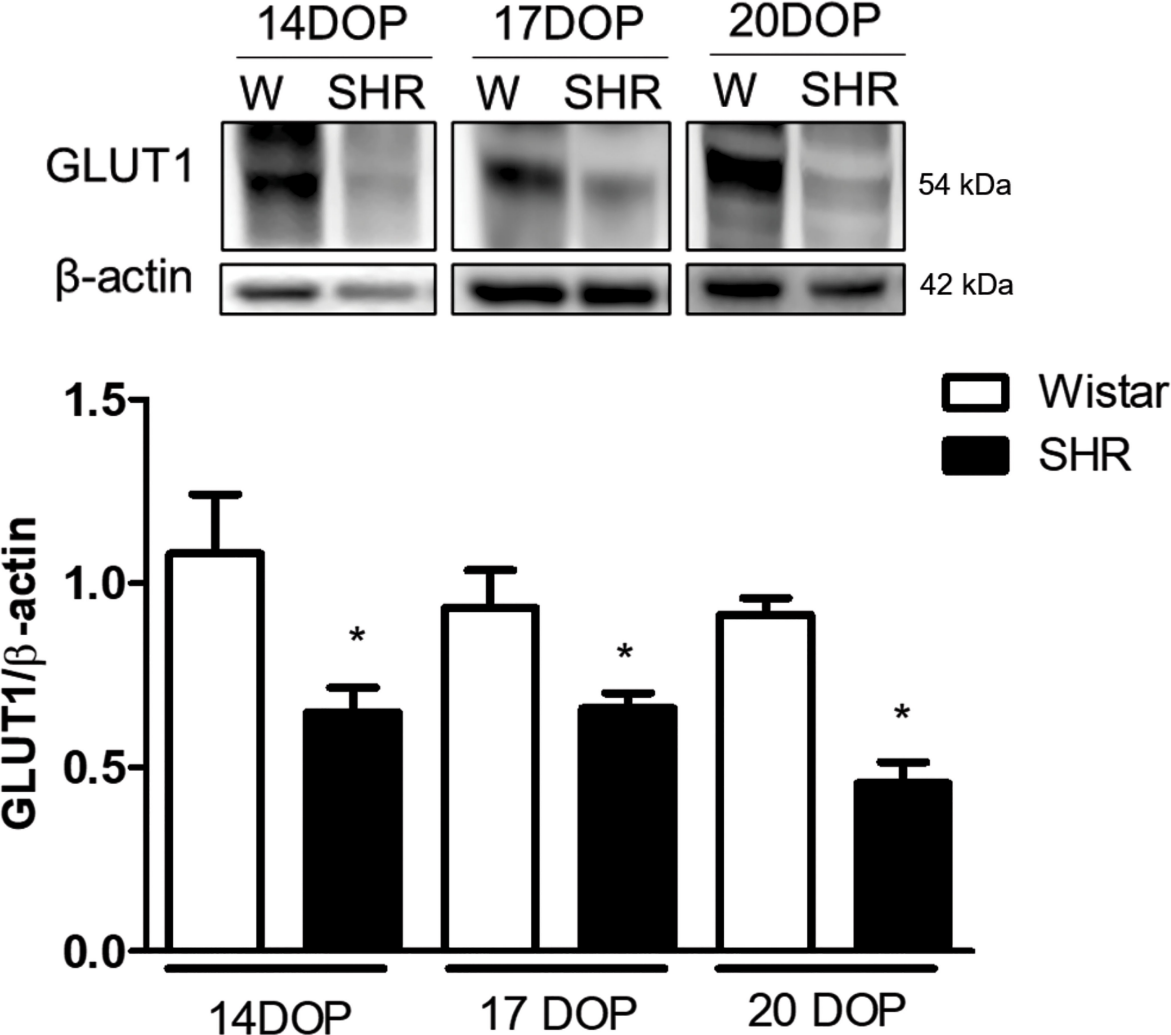
Reduced placental GLUT1 expression in SHR at 14, 17 and 20 DOP. Upper representative picture of western blot membrane of placental GLUT1 expression in Wistar and SHR at 14, 17 and 20 DOP. Bar graph showing the GLUT1 expression in Wistar and SHR at 14, 17 and 20 DOP; n=6 each group. Values are presented as means ± SEM, and data were analyzed by one‐way ANOVA, followed by Tukey post-test. *p < 0.05 vs Wistar at respective DOP. Protein expression was individually determined and corrected by β−actin expression.

Moreover, GLUT1 expression was found to be decreased in placentas from SHR at 14, 17, and 20 DOP when compared to Wistar rats (**Figure 5**).

## Discussion

In this study, we sought to investigate the relationship between protein *O*-GlcNAcylation, placental glucose availability, and fetal growth throughout the pregnancy affected by hypertension. Our findings showed that hypertension interestingly promotes alterations in the protein *O*-GlcNAc profile throughout pregnancy that are associated with placental structure-function alterations and impaired fetal growth, due to decreased placental glucose availability in SHR.

In the present study, SHR presented decreased fetal weight at 14, 17, and 20 DOP with an increasing percentage of SGA fetuses. Near-term, 100% of all the fetuses from SHR were SGA, indicating intrauterine growth retardation. The growth of a healthy baby relies on a healthy placenta, and functional or structural defects of the placenta impact fetal growth (21). In this study, the placental weight increased throughout pregnancy, and near-term placentas from SHR were found to be significantly heavier, compared to Wistar. A bigger placenta could indicate a compensatory mechanism to ensure proper nutrient supply to the growing fetus due to uteroplacental hypoperfusion resulting from increased blood pressure (22). Moreover, the fetal/placental weight ratio was found to be decreased in SHR at 17 and 20 DOP, indicating a loss of placental efficiency from the 17th DOP until term. In rodents, placental efficiency can be estimated by the grams of fetus produced per gram of placenta (23), and a reduced fetal/placental weight ratio may indicate a dysfunctional placenta because fetuses do not grow properly despite an enlarged placenta. These findings are in accordance with previous studies that already described fetal and placental parameters in this animal model (24–26). However, the previous reports did not show the relation between *O*-GlcNAc, placental development, and fetal growth, as reported here.

*O*-GlcNAcylation is known to regulate the function of more than 4000 proteins, therefore contributing to the appropriate modulation of cellular responses and adaptation to cellular stress (27). Several stages of embryonic development during pregnancy have been shown to depend on the *O*-GlcNAc cycling, including placentation (28). Protein *O*-GlcNAcylation has been implicated in embryonic development once the OGT enzyme was determined to be essential for embryonic stem cell viability and OGT depletion was related to embryonic lethality (29, 30). Lately, *O*-GlcNAc was found to promote trophectoderm differentiation into invasive trophoblast, a pattern required during embryo implantation (31). These data could relate to our findings once SHR presented an increased number of pre-implantation losses along with reduced placental *O*-GlcNAc expression since 14 DOP. We speculate that protein *O*-GlcNAcylation may be reduced in the early stages of embryonic development as a consequence of hypertension in these animals.

To better understand the importance of *O*-GlcNAc in placental tissue, a recent study documented approximately 750 *O*-GlcNAcylated proteins in trophoblast and fetal capillaries within the villous of the human placenta (32). Here, we show structural alterations of the placenta in SHR at 14, 17, and 20 DOP. A reduced Jz area of the placenta was found on all DOPs analyzed, and the Lz showed a compacted area with thicker wall vessels and vascular congestion. Interestingly, immunohistochemistry showed reduced protein *O*-GlcNAcylation on both Jz and Lz of the placentas from SHR. The Jz contains three main cell types (spongiotrophoblast cells, trophoblast giant cells, and glycogen cells), and constitutes the main endocrine compartment of the placenta (33). The Lz comprises the entire placental exchange area and is responsible for transport in which maternal and fetal blood circulations come into close contact without mixing (34). Structural defects in both Jz and Lz during placentation impair fetal development (35, 36). In this regard, a few proteins and transcription factors that are important for the development of the placental tissue were found to be *O*-GlcNAcylated. For example, the hypoxia-inducible factor-1 alpha (HIF-1α), essential for placental vascular development, is a target for the *O*-GlcNAc cycling enzymes OGA and OGT, playing a critical role in HIF-1α stabilization (37). Another example is the histone H2A, which is highly expressed in the early mouse placenta (38), where its *O*-GlcNAcylation was found to be important for trophoblast differentiation and placental development (39). Finally, the specific protein 1 (SP1), a transcription factor involved in placental trophoblast invasion and migration, is also a target for *O*-GlcNAc (40).

As mentioned before, HBP and *O*-GlcNAcylation have been established as nutrient sensor signaling pathways (10). Therefore, *O*-GlcNAc pathway regulation is critical for growth signaling of the human placenta, and OGT has been elicited as a primary nutrient sensing protein, involved in glucose and amino acid utilization, and as a biomarker of cellular stress (11, 41). Hypertension in pregnancy is known to be associated with placental hypoperfusion (42, 43) and hypoxia was shown to decrease *O*-GlcNAcylation. Here, we observed reduced placental protein *O*-GlcNAcylation in all DOP analyzed concomitantly with reduced OGT and OGA expression in 14 DOP in SHR. These data, combined with the reduced PAS stain, could indicate nutrient stress in the early placenta as a consequence of decreased placental glucose uptake during chronic hypertension. In fact, disruption in this nutrient-sensing pathway is related to placental insufficiency and fetal growth restriction. Moreover, we believe that the decreased OGA expression observed here may be a compensatory mechanism to raise protein *O*-GlcNAc levels. Furthermore, while maternal nutrition influences significant placental changes that affect fetal growth, the majority of cases of fetal growth restriction result from changes in placenta function that result in the dysfunctional transport of nutrients, especially glucose and amino acids, which have been linked to OGT sensing pathways (44, 45).

Thus, we decided to investigate glucose transport availability in the placentas of hypertensive rats. In rodents, glucose is stored in the form of glycogen cells grouped in clusters in the Jz of the placenta. PAS analysis showed a reduced number of glycogen cells per mm^2^ of Jz in placentas from SHR at 14, 17, and 20 DOP when compared to Wistar rats. On day 17, a substantial amount of glycogen cells was found in both SHR and Wistar placentas when compared to the other days. This indicates that glycogen storage peaks at this period. Previous studies have also shown a peak in placental glycogen stores between 15.5 and 18.5 days (46, 47). Moreover, reduced glycogen stores in placentas of stroke-prone SHR have been described previously at 18 DOP, compared to the Wistar-Kyoto strain (48) and this was associated with inadequate uterine artery remodeling and uteroplacental blood flow in these animals.

Fetal growth and development require glucose as the primary nutrient, which is transported across the placenta through facilitated diffusion by the glucose transporter family. GLUT1 was described as the principal glucose transporter in the placenta (49), and its expression was found to increase throughout gestation in humans (45). In this study, placentas from SHR presented decreased GLUT1 expression at 14, 17, and 20 DOP when compared to Wistar. Thus, nutrient flux from glucose, fatty acid, and nucleotide metabolism in the placenta is expected to impact OGT’s enzymatic activity in the placenta. Less is known about the role of OGT regulation of nutrients in fetal growth restriction. However, OGT-deficient placentas and those from growth-restricted fetuses contain diminished levels of GLUT1 receptors (50). GLUT1 protein expression was found to be down-regulated, and glucose transport activity was decreased in placentas from pregnancies affected by preeclampsia (51). Recently, a study showed that in addition to reduced GLUT1 in the decidua of patients with severe preeclampsia, GLUT1 deficiency may trigger aberrant glycolysis, thereby leading to poor decidualization and subsequent impaired placental development (52). Furthermore, a recent study showed that *O*-GlcNAcylation mediates the regulation of the water channel aquaporin 3 (AQP3) and that both elevated *O*-GlcNAcylation and AQP3 increase glucose uptake *via* GLUT1 (53). Interestingly, we have previously described that SHR presents a lack of placental AQP3 expression and that AQP3 is important for trophoblast cell migration, a crucial step during placentation (54). Curiously, loss of AQP3 in the placentas was shown to induce growth restriction in mice (55). *O*-GlcNAcylation was found to regulate GLUT1 through c-Myc (56) and AQP3 through SP1 (53). Together, our findings make a significant contribution to our understanding of how protein *O*-GlcNAcylation affects fetal growth and placental function during hypertension.

Finally, the data we presented establish protein *O*-GlcNAcylation cycling as a nutrient sense signaling and cellular stress biomarker of placental dysfunction and impaired fetal growth during hypertension in pregnancy. Moreover, we showed reduced *O*-GlcNAc in the two main functional parts of the placenta, which could be related to structural and functional derangements of the placenta in SHR.

## Data availability statement

The original contributions presented in the study are included in the article/supplementary material. Further inquiries can be directed to the corresponding author.

## Ethics statement

The animal study was reviewed and approved by Ethics Committee on the Use of Animals of the Federal University of Mato Grosso (CEUA-Araguaia).

## Author contributions

RRPJ: designed the hypothesis, performed research and analyzed data, and wrote the manuscript. RAF: performed research and data curation. VDJ: performed research and data curation. SSM: supervision, data curation, and revision of the manuscript. VVL: supervision, data curation, and revision of the manuscript. FG: designed the hypothesis, supervision, project administration, revision of the manuscript, and funding acquisition. All authors contributed to the article and approved the submitted version.

## Funding

This work was supported by the Fundação de Amparo à Pesquisa do Estado de Mato Grosso (FAPEMAT, 443/2022 to FG); Conselho Nacional de Desenvolvimento Científico e Tecnológico (CNPq, 406974/2021-7 to FG, and 141502/2020-7 Scholarship to RRPJ).

## Acknowledgments

The authors thank the Biomedical Research Center, School of Medicine, Universidad de Valparaíso, Valparaíso, Chile, and the Laboratory of Vascular Biology and Histopathology of the Federal University of Mato Grosso, Barra do Garças, Brazil.

## Conflict of interest

The authors declare that the research was conducted in the absence of any commercial or financial relationships that could be construed as a potential conflict of interest.

## Publisher’s note

All claims expressed in this article are solely those of the authors and do not necessarily represent those of their affiliated organizations, or those of the publisher, the editors and the reviewers. Any product that may be evaluated in this article, or claim that may be made by its manufacturer, is not guaranteed or endorsed by the publisher.
